# Can Online Academic Integrity Instruction Affect University Students’ Perceptions of and Engagement in Academic Dishonesty? Results From a Natural Experiment in New Zealand

**DOI:** 10.3389/fpsyg.2021.569133

**Published:** 2021-02-17

**Authors:** Jason Michael Stephens, Penelope Winifred St John Watson, Mohamed Alansari, Grace Lee, Steven Martin Turnbull

**Affiliations:** The University of Auckland, Auckland, New Zealand

**Keywords:** natural experiment, tertiary students, academic dishonesty, online instruction and learning, academic integrity initiatives

## Abstract

The problem of academic dishonesty is as old as it is widespread – dating back millennia and perpetrated by the majority of students. Attempts to promote academic integrity, by comparison, are relatively new and rare – stretching back only a few hundred years and implemented by a small fraction of schools and universities. However, the past decade has seen an increase in efforts among universities to promote academic integrity among students, particularly through the use of online courses or tutorials. Previous research has found this type of instruction to be effective in increasing students’ knowledge of academic integrity and reducing their engagement in academic dishonesty. The present study contributes to this literature with a natural experiment on the effects of the Academic Integrity Course (AIC) at The University of Auckland, which became mandatory for all students in 2015. In 2012, a convenience sample of students (*n* = 780) had been asked to complete a survey on their perceptions of the University’s academic integrity polices and their engagement in several forms of academic dishonesty over the past year. In 2017, the same procedures and survey were used to collect data from second sample of students (*n* = 608). After establishing measurement invariance across the two samples on all latent factors, analysis of variance revealed mixed support for the studies hypotheses. Unexpectedly, students who completed the AIC (i.e., the 2017 sample) reported: (1) significantly lower (not higher) levels of understanding, support, and effectiveness with respect to the University’s academic integrity policies; (2) statistically equivalent (not higher) levels of peer disapproval of academic misconduct, and; (3) significantly higher (not lower) levels of peer engagement in academic misconduct. However, results related to participants’ personal engagement in academic misconduct offered partial support for hypotheses – those who completed the AIC reported significantly lower rates of engagement on three of the eight behaviors included in the study. The implications and limitations of these findings are discussed as well as possible future directions for research.

## Introduction

The problem of academic dishonesty is an ancient one, dating back millennia (Lang, 2013). For the past 50 years, the problem has been epidemic – with the majority of students reporting that they have cheated, plagiarized, or otherwise behaved dishonestly at least once in the past year (for a review, see Murdock et al., 2016). By comparison, attempts to promote academic integrity are relatively new, stretching back only a few hundred years. The past decade, in particular, has seen an increase in efforts among universities to promote academic integrity among students. These efforts include face-to-face workshops and online courses as well as blended learning approaches (Stoesz and Yudintseva, 2018). The most common (and growing) approach appears to be requiring (or at least encouraging) incoming students to complete a short, web-based course or tutorial on academic integrity.

Previous research has shown that such tutorials can be effective not only in increasing students’ knowledge of academic integrity (e.g., Curtis et al., 2013; Cronan et al., 2017) but also reducing their engagement in academic dishonesty (e.g., Belter and du Pré, 2009; Dee and Jacob, 2010; Owens and White, 2013; Zivcakova et al., 2014). However, there are important differences in how these academic integrity tutorials have been implemented and assessed in the existing research and the way in which they are being used (and often left unassessed) by an increasing number of universities. At the University of Auckland, for example, all new students are required to complete the Academic Integrity Course (AIC) during their first semester. Like many of the tutorials studied in previous research, the AIC is a short online course that students are expected to complete by themselves in their own time.

However, unlike the tutorials in extant studies (which were completed by students as a requirement in one of their academic courses), the AIC is not connected to any academic courses or programs. Moreover, existing studies also limited the assessment of tutorial effects to the specific course in which the tutorial was required; whereas a comparable assessment of the AIC (and other courses like it) would necessitate looking for effects across all applicable courses. Finally, in prior research, assessment of the tutorial was completed either immediately following completion of the tutorial or within weeks thereafter. In contrast, an assessment of an existing tutorial or course (like the AIC) may involve students who completed it years earlier.

The present investigation seeks to broaden the research on academic integrity tutorials with a natural experiment on the effects of the AIC. That is, by assessing the impact of the course “in the everyday (i.e., real life) environment of the participants, [where] the experimenter has no control over the IV as it occurs naturally in real life” (McLeod, 2012), the present investigation extends the methodological approaches that have been used to assess tutorial effects. In doing so, the present study hopes to offer important contributions to the growing literature on academic integrity tutorials, particularly concerning the extent to which the positive effects previously reported can be replicated under different conditions.

## Literature Review

### A Long History of Academic Dishonesty

Academic dishonesty (also termed “academic misconduct” or, most succinctly, “cheating”) has a long history. Although the first documented report of cheating pertained to sport rather than academia, the contextual elements typically at play in decisions to cheat in the two domains are similar – extended periods of intense preparation leading up to a comparatively brief moment of competition (often against one’s peers, if not the clock or other criteria as well) resulting in immense pressure to perform near perfection (Lang, 2013). Such were the circumstances of the Thessalian boxer, Eupolos, who was apprehended while trying to bribe his opponents during the early fourth century Olympic Games in Greece (Guttman, 2004; Spivey, 2004).

The setting of the first known case(s) of academic cheating – involving China’s stringent civil service examinations during the Sui dynasty on the seventh century – was not much different. The exams required a thorough knowledge of Confucius’ works, skill in poetry writing in Confucian style, and memorization of the complete Imperial documents on education. Years of preparation were necessary, and failure (the lot of most candidates) resulted in vastly reduced life opportunities, misery for many, and suicide for some. Cheating abounded, with the use of model answers secreted about candidates’ persons, and bribery as examples (Lang, 2013).

Other records of academic dishonesty – dating from the 1760s to the present day – confirm that young people who typically displayed impeccable honor in other aspects of their lives have engaged in cheating in academic contexts (e.g., Hartshorne and May, 1928; Bertram Gallant, 2008). The first large-scale study of cheating occurred in the United States in the 1960s and indicated that 75% of United States tertiary students had cheated at least once in their academic careers (Bowers, 1964). Remarkably, this very high percentage has only fluctuated modestly over the past five decades – the majority of tertiary students (the world over, wherever asked) report having cheated in the past year (e.g., Lupton et al., 2000; Lupton and Chapman, 2002; McCabe, 2005; Stephens et al., 2010; Ma et al., 2013).

While digital technologies have made some forms of dishonesty much quicker and easier (from do-it-yourself cut-n-paste plagiarism to paying a “shadow scholar” to write a bespoke paper for you), they are not the reason *why* students cheat. A sober look at our evolution and ontogeny make it clear that cheating is “natural and normal” even if – with respect to academic dishonesty – “unethical and evitable” (for an overview of evidence, see Stephens, 2019).

With this mind, the important question is not who is most likely to cheat and under what circumstances, but rather what conditions are most likely to mitigate cheating. If the history of cheating has a lesson to teach us about dishonesty, it is less about the weakness of humans and more about the power of circumstances. It is the same lesson that contemporary empirical research teaches us – contextual factors outweigh individual characteristics in explaining variance in cheating behavior (e.g., Leming, 1978; McCabe and Trevino, 1997; Eisenberg, 2004; Day et al., 2011). Among the contextual factors that seem to matter most in reducing academic dishonest are efforts to promote academic integrity.

### The (Relatively) Brief History of Promoting Academic Integrity

Punishment and prevention are distinct, and it is a mistake to believe that threats of former (if only severe enough) equates with the latter. As described above, academic dishonesty dates back millennia and is epidemic today. This despite the fact that early “deterrents” for cheating included flogging, public ridicule, stripping of academic credentials, banishment from one’s hometown, and even death (Lang, 2013). The history of using severe punishment as both penalty and prevention is as long as the history of cheating itself, and that history has taught us that it is neither ethical nor effective. In short, punishment is not a very good teacher – it arrives late and is quite primitive (seeking to condition a basic stimulus-response association rather than a mindful understanding).

Efforts to *promote* academic integrity – attempts to advance a conscious understanding and active commitment to honesty in one’s scholarly endeavors – are comparatively recent (relative, that is, to police and punish approaches of deterrence). The first such effort dates back to 1736 with the establishment of the Honor Code at The College of William and Mary^1^. The code is run by students, who are responsible for its administration and maintenance, including the adjudication of suspected violations. Today, there are over a hundred colleges and universities (mostly in North America and most private, highly selective institutions) with honor codes. Nonetheless, research has shown significant differences in academic dishonesty between honor code institutions and their non-code counterparts (McCabe and Trevino, 1993; McCabe et al., 1999; Schwartz et al., 2013). In their oft-cited study, McCabe and Trevino (1993) found that only 29% of students at the former reported cheating in the past year compared to 53% at the latter. More importantly, research also suggest the lower level of cheating at honor code institutions is attributed to the “culture of integrity” they engender and not the threat of punishment (McCabe et al., 1999).

While honor codes, and other systems-based, multi-level approaches to creating cultures of integrity (e.g., Bertram Gallant, 2011; Stephens, 2015) offer the most comprehensive approach to promoting academic integrity, they require more time and commitment than most institutions are willing or able to invest. However, the past decade has seen numerous high profile cases of cheating in the headlines (e.g., Perez-Pena and Bidgood, 2012; Visentin, 2015; Medina et al., 2019) and with them an increase in number of colleges and universities seeking to do something, even if small(er) in scope and scale. At a minimum, these efforts include the adoption of online methods of self-checking for plagiarism – allowing students to see their “mistakes” and learn from them (Walker, 2010). More pro-active still is the creation of face-to-face workshops, online modules, or blended approaches that provide opportunities to develop the knowledge and skills needed to achieve with integrity *before* mistakes are made (e.g., York University^2^).

The adoption of such approaches has spread widely and numerous universities around the world now require their students to complete a course or tutorial on academic integrity during or shortly after matriculation. Australia appears to be leading the way in terms of implementing a national framework and requirements. In the wake of several high profile cheating scandals (e.g., McNeilage and Visentin, 2014), the Tertiary Education Quality and Standards Agency (the national quality assurance and regulatory agency for education) revised its Higher Education Standards Framework to include four broad requirements (of all tertiary education providers) related to academic and research integrity. The third requirement is “to provide students and staff with guidance and training on what constitutes academic or research misconduct and the development of good practices in maintaining academic and research integrity” (Universities Australia, 2017, p. 1). As a result, over half of all Australian universities offer (but not always require students to complete) online tutorials on academic integrity. Despite similar high profile cheating scandals in New Zealand (e.g., Jones, 2014; Weeks, 2019), there is not (yet) a comparable framework or set of requirements in place there; and, perhaps not incidentally, only one university in the country that requires its students to complete a course on academic integrity (i.e., the AIC at the University of Auckland described below).

Empirical research over the past decade suggest such approaches can be effective not only increasing knowledge and attitudes (e.g., Curtis et al., 2013; Cronan et al., 2017) but also reducing dishonesty (e.g., Belter and du Pré, 2009; Dee and Jacob, 2010; Owens and White, 2013; Zivcakova et al., 2014). For example, results from field-based quasi-experimental (Belter and du Pré, 2009) and experimental (Dee and Jacob, 2010) research showed that completion of online tutorials related to plagiarism greatly reduced its occurrence – by 75.4% in the former study and an estimated 41% in the latter. However, as noted in the introduction, there is no evidence to date that the effects of these tutorials extend beyond the immediate context (i.e., the academic course) in which they are implemented (Stoesz and Yudintseva, 2018). Additionally, the empirical research on the effects of academic integrity instruction has yet to include any delayed testing. To date, all of the studies have assessed the effects of such instruction immediately or shortly after it was delivered.

In short, it is not clear yet if the learning and behavioral changes associated with these tutorials transfers to other contexts (i.e., courses beyond the one in which the learning occurred) or across longer periods of time (i.e., greater than a few minutes or few weeks). There are both theoretical and empirical reasons to believe that the more we move away – in place and time – from something we’ve learned, the less likely we are to recall or utilize that learning. Specifically, from a learning transfer perspective (e.g., Perkins and Salomon, 1992), extant studies have required *near transfer* of learning (i.e., demonstration of learning in the same or highly similar context) and not *far transfer* (i.e., impact in a context outside of or remote from the context of learning). Similarly, research on recency effects has provided strong evidence that are better able to remember recent experiences than those that happened further back in time (e.g., Morgeson and Campion, 1997; Jones and Sieck, 2003). The present investigation on attitudinal and behavioral differences associated with completing the AIC extends the boundaries (contextual and temporal) of existing research on the effect of academic integrity instruction.

### The Academic Integrity Course at the University of Auckland

Developed by professional library staff at The University of Auckland (UOA) in 2013, the Academic Integrity Course (AIC) has been compulsory for all matriculating students since 2015. Students are enrolled in the AIC during their first term (semester or quarter), and failure to complete the course results in automatic re-enrolment for the next term. Although a student may forestall completion of the course, it is required for the conferral of any degree, diploma or certificate. According to a university official (personal communication), “most students” complete the AIC within their first of study at the university.

**FIGURE 1:**
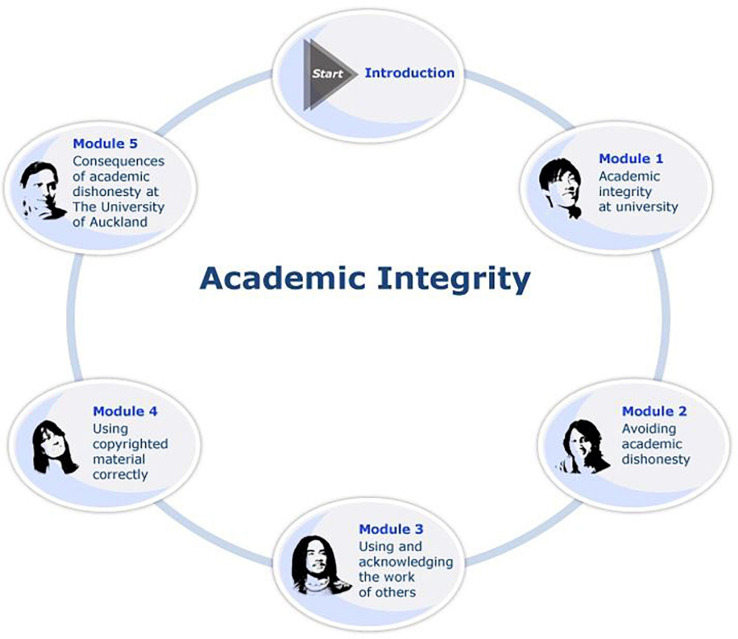
The Academic Integrity Course (AIC) at the University of Auckland. The AIC is a self-paced online course in which students are automatically enrolled during their first semester at the university.

As depicted in Figure 1, the AIC is comprised of five modules course, each comprised of readings that teach students about the different facets of academic integrity and each with a test at the end that students must finish. The AIC is an online and self-paced, allowing students to complete the modules and tests in their own time. The modules and tests can be completed in any order the student prefers, but they must achieve 100% on all five tests (with unlimited attempts to do so). Completion time for the whole course is between one and two hours.

Finally, with respect to its goals, the AIC was “designed to increase student knowledge of academic integrity, university rules relating to academic conduct, and the identification and consequences of academic misconduct^3^.” As described by the University’s Deputy Vice-Chancellor (Academic) John Morrow, the AIC is “not about dishonesty as such, it’s about students learning how they can use printed and published resources in an effective way in their own work” (as quoted in van Beynen, 2015). For a topical outline of the AIC, please see Appendix A.

### The Present Investigation

The primary purpose of the present investigation was to test the effects of the AIC on students’ perceptions of and engagement in academic dishonesty. Specifically, compared to students who did not do so, it was hypothesized that students who completed the AIC would report:

H_1_: greater perceived understanding, support for, and effectiveness of university policies related to academic integrity;
H_2_: greater perceived peer disapproval of academic dishonesty;
H_3_: lower levels of perceived peer engagement in academic dishonesty; and
H_4_: lower levels of personal engagement in three types of academic dishonesty (i.e., assignment cheating, plagiarism, and test/exam cheating).

The foregoing hypotheses are based prior research indicating that tutorials (similar to the AIC) have been shown to increase students’ knowledge of academic integrity (e.g., Curtis et al., 2013; Cronan et al., 2017) and reduce their engagement in academic dishonesty (e.g., Belter and du Pré, 2009; Dee and Jacob, 2010; Owens and White, 2013; Zivcakova et al., 2014). However, as described above, the literature on learning transfer (e.g., Brown, would suggest that any effects in the present study (with its demand for far rather than near transfer) are likely to smaller than those found in previous research.

Finally, based on the existing literature on recency effect, it hypothesized (H_5_) that all of the foregoing effects would be stronger among participants who completed the AIC within the past month and weakest among those who completed it two or more years ago.

## Materials and Methods

The effects of the AIC on students’ perceptions of and engagement in academic dishonesty were assessed using a quasi-experimental research design; namely, a natural experiment. Unlike a true experiment, a natural experiment is a type of observational study in which individuals (or groups of them) are *naturally* (i.e., determined by nature or other factors beyond the control of researchers) exposed to control and experimental conditions. The processes determining participants’ exposures only *approximates* random assignment. In the present investigation, survey data related to students’ academic integrity perceptions and behavior was collected as a part of course-based research exercise carried out in 2012 and 2017. When the AIC became mandatory for all students in 2015, it rendered (*approximately*) the 2012 participants a control or comparison group (i.e., AIC non-existent) and the 2017 participants an experimental or treatment group (i.e., AIC compulsory). To be clear, the data collect in 2012 data were not collected for the purposes of publication. It was not until 2016 that the authors thought about using the 2012 data as part of a natural experiment to test the effects of the AIC; deciding to replicate the research exercise (and data collection associated with it) in 2017. This research was approved by The University of Auckland Human Participants Ethics Committee in 2012 (Reference Number 2009/C/026) and again in 2017 (Reference Number 019881).

### Procedures

As noted above, data for this investigation were collected under the auspices of a course-based research project. The course was an advanced level undergraduate course in educational psychology that required students take on the role of researchers. Specifically, all students enrolled in the course in 2012 (*n* = 108) and 2017 (*n* = 48) were asked to recruit 8 (in 2012) or 15 (in 2017) other UOA students (not enrolled in the course) to complete a short anonymous survey (detailed below in Measures). In 2012, 93.5% of students (101 of the 108 enrolled) completed the assignment and, in 2017, 95.8% (46 of 48) did so. Importantly, with the exception of the number of participants students were asked to recruit for the study (owing to the different enrolment numbers), the instructions provided to students and the sampling procedures used by them were identical in 2012 and 2017. Please see Appendix B for a copy of the assignment instructions provided to students.

### Participants

In 2012, the 101 students who completed the course research activity recruited a total of 803 students to participate in the study; 23 of whom (2.9%) were subsequently removed from the sample for missing data. In 2017, the 46 students who completed the same activity recruited a total of 674 students to participate in the study; 31 of whom (4.6%) were subsequently removed from the sample for missing data and an additional 41 (6.1%) because they indicated that they had not yet completed the AIC. Thus, the final sample included 1,388 university students: 780 in 2012 and 608 in 2017. As detailed in Table 1, the majority of participants in both cohorts were females (57.7% and 57.6% in 2012 and 2007, respectively) and drawn from all eight faculties within the university. Importantly, although percentages varied slightly between cohorts, the differences in observed versus expected counts (given marginal frequencies) were not statistically significant for either gender (χ^2^ = 1.07, *p* = 0.786) or faculty (χ^2^ = 12.49, *p* = 0.093).

**TABLE 1:** Participants’ gender and faculty by cohort: frequencies and percents.

	2012 Cohort		2017 Cohort		Total sample	
Variable	*n* = 780	%	*n* = 608	%	*N* = 1388	%
**Gender**						
Female	450	57.7	350	57.6	800	57.6
Male	322	41.3	243	40.0	565	40.7
Missing	8	1.0	15	2.5	23	1.7
**Faculty**						
Arts	229	29.4	169	27.8	398	28.7
Science	157	20.1	122	20.1	279	20.1
Business	125	16.2	118	19.4	243	17.5
Engineering	62	7.9	76	12.5	138	9.9
Medical	64	8.2	40	6.6	104	7.5
Law	43	5.5	36	5.9	79	5.7
Education	43	5.4	24	3.9	67	4.8
NICAI	11	1.4	10	1.6	21	1.5
Missing	46	5.9	13	2.1	59	4.3

*There were no statistically significant between-group differences by either Gender (χ^2^ = 1.07, *p* = 0.786) or Faculty (χ^2^ = 12.49, *p* = 0.093).*

### Measures

The survey used in the present investigation was comprised of measures adapted from the Academic Motivation and Integrity Survey (AMIS; Stephens and Wangaard, 2013). In addition to the measures and items, participants in 2012 and 2017 were asked to report their sex (where 0 = Female, 1 = Male, and 3 = Other/prefer not to say) and faculty affiliation (e.g., Arts, Sciences, Business, Engineering).

#### Perceptions

Students’ perceptions of their peers’ attitudinal and behavioral norms related to academic integrity were assessed with three measures:

#### USE of Academic Integrity Policies

Participants’ perceptions of the understanding, support and effectiveness (USE) the university’s policies related to academic integrity were assessed with a 5-point Likert-type scale ranging from 1 (very low) to 5 (very high). Specifically, participants used this scale to rate three statements: “The average student’s understanding of policies concerning cheating,” and “The average student’s support of policies concerning cheating,” and “The effectiveness of these policies.”

#### Peer Disapproval of Academic Dishonesty

Participants’ perceptions related to peer disapproval of academic dishonesty was assessed with a 5-point Likert-type scale that ranged from 1 (not at all) to 5 (very strongly). Specifically, participants were asked to report about how strongly their peers would disapprove if they knew they had engaged in three types of academic dishonesty: “homework cheating,” “plagiarism,” and “test or exam cheating.”

#### Peer Engagement in Academic Dishonesty

Participants’ perceptions related to peer engagement in academic dishonesty were assessed with a 5-point Likert-type scale ranging from 1 (never) to 5 (almost daily). Specifically, students were asked to report about how often, during the past year, they “thought” other students had engaged in three types of cheating behavior: “copying each other’s homework,” “plagiarism,” and “cheating on tests or exams.”

#### Behaviors

Personal engagement in academic dishonesty was assessed with a 5-point Likert-type scale that ranged from 1 (never) to 5 (almost daily). Specifically, participants were asked to use that scale to indicate how often they had engaged in eight “academic behaviors” that comprised three types of academic dishonesty:

#### Assignment Cheating

1. Copied all or part of another student’s work and submitted it as your own.
2. Worked on an assignment with others when the instructor asked for individual work.

#### Plagiarism

3. From a book, magazine, or journal (not on the Internet): Paraphrased or copied a few sentences or paragraphs without citing them in a paper you submitted.
4. From an Internet Website: Paraphrased or copied a few sentences or paragraphs without citing them in a paper you submitted.

#### Test or Exam Cheating

5. Used unpermitted notes or textbooks during a test or exam.
6. Used unpermitted electronic notes (stored in a PDA, phone or calculator) during a test or exam.
7. From a friend or another student: Copied from another’s paper during a test or exam with his or her knowledge.
8. Used digital technology such as text messaging to “copy” or get help from someone during a test or exam.
9. Got questions or answers from someone who has already taken a test or exam.

Finally, the 2017 survey included one extra question, which asked participants to indicate when they completed the AIC (where 1 = Within past 3 months, 2 = 4 to 6 months ago, 3 = 7 to 12 months ago, 4 = 1 to 2 years ago, 5 = 2 or more years ago, and 6 = I have not yet completed it).

### Data Analyses

Data were first screened for missing or invalid responses, and then subjected to confirmatory factor analysis (CFA) to confirm the structure and fit of the two three-factor measurement models. Based on recommendations by Hu and Bentler (1999) and Ullman and Bentler (2003), normed chi-square values and several other indices were used to determine model fit, where χ^2^/*df* < 3.0, CFI > 0.95, TLI > 0.95, RMSEA < 0.06, and SRMR < 0.05) for a “good” fit and χ^2^/*df* < 5.0) CFI > 0.90, TLI > 0.90, RMSEA < 0.08, and SRMR < 0.08) for an “acceptable” fit. Given the need to compare cohorts of participants sampled 5 years apart, multi-group confirmatory factory analysis (MGCFA) was employed to test for measurement invariance across groups/time (to ensure the psychometric equivalence of the constructs). Based on recommendations by Chen (2007), change in CFI, RMSEA, and SRMR values were used to determine the level of invariance achieved: ΔCFI of < −0.01 and ΔRMSEA of < 0.015 for each successive level, and ΔSRMR of < 0.030 for metric invariance and < 0.015 for scalar or residual invariance. After establishing acceptable model fit and measurement invariance, Cronbach’s alphas were calculated to assess the internal consistency of the six factors and Pearson correlation coefficients to assess their convergent and discriminant validity. Finally, ANOVA and cross-tabulations with Chi-square analyses were employed to test study hypotheses. All analyses were conducted using version 25 of SPSS and its AMOS programme.

## Results

Results from the CFA and MGCFA and are reported first, followed by the descriptive statistics of and intercorrelations among the six latent factors. Subsequently, results pertaining to the four hypotheses are described.

### Confirmatory Factor Analysis of the Two Three-Factor Measurement Models

Confirmatory factor analysis was used to test the validity of the two three-factor measurement models for both cohorts of participants. That is, before proceeding to MGCFA to test for measurement invariance, CFA was conducted to ensure that the factor structure (configuration of paths) was equivalent across cohorts. As detailed in Table 2, whether combined or tested independently, the data from both cohorts offered a good (or at least acceptable) fit to both models. The only exception was Model 1.3 for Behaviors with normed Chi-square (χ^2/^*df*) value of 5.02 and RMSEA value of 0.082 (both slightly above the recommended threshold values of 5.00 and 0.080, respectively). Examination of the modification indices suggested freeing the covariance between two error terms (i.e., the two forms of digital test cheating). Doing so significantly improved the model fit (χ^2/^*df* = 2.00, TLI = 0.989, CFI = 0.994, RMSEA = 0.040, and SRMR = 0.024). However, given the sensitivity of χ^2^ in large samples, and that the other indices (i.e., the TLI, CFI, and SRMR) indicated a “good” fit, the decision was deem the model acceptable as hypothesized (without freeing the covariance between error terms).

**TABLE 2:** Model fit statistics for the two three-factor measurement models of perceptions and behaviors.

Model	χ^2^	*df*	χ^2^/*df*	TLI	CFI	RMSEA (90% CI)		SRMR
**3-Factor Model of Perceptions**								
Model 1.1 – Full Sample	104.18	24	4.34	0.974	0.983	0.049	(0.040–0.059)	0.049
Model 1.2 – 2012 Cohort	71.85	24	2.99	0.971	0.981	0.051	(0.037–0.064)	0.051
Model 1.3 – 2017 Cohort	88.60	24	3.69	0.954	0.969	0.067	(0.052–0.082)	0.062
**3-Factor Model of Behaviors**								
Model 1.1 – Full Sample	72.51	17	4.27	0.984	0.990	0.048	(0.037–0.060)	0.025
Model 1.2 – 2012 Cohort	64.77	17	3.81	0.976	0.985	0.060	(0.045–0.076)	0.026
Model 1.3 – 2017 Cohort	86.99	17	5.12	0.952	0.971	0.082	(0.066–0.010)	0.036

*Full Sample *N* = 1388; 2012 Cohort *n* = 780; 2017 Cohort *n* = 608.*

**FIGURE 2:**
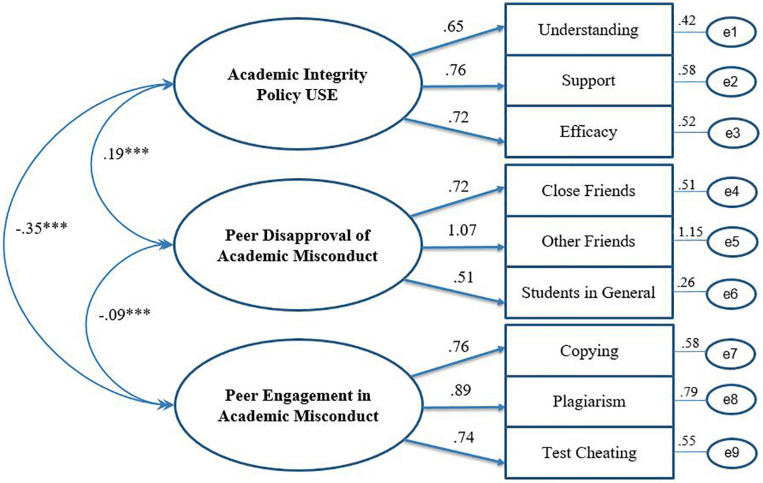
Standardized estimates of the three-factor measurement model of participants’ perceptions related to academic integrity policies and peer norms. Model 1.1 – Full Sample. ^∗∗∗^*p* < 0.001.

**FIGURE 3:**
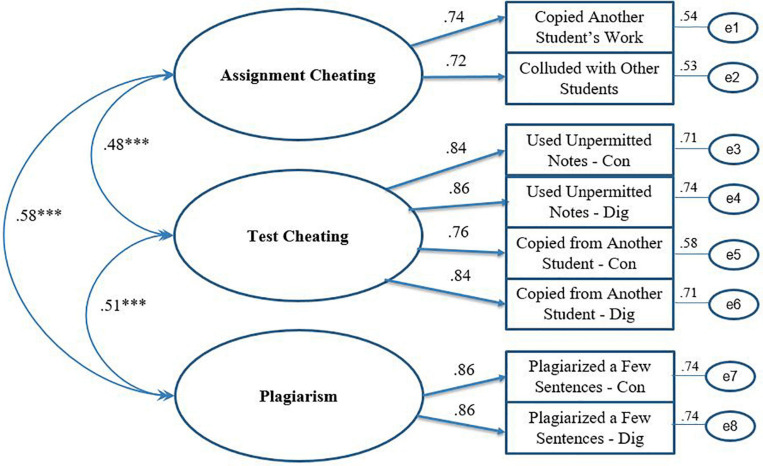
Standardized estimates of the three-factor measurement model of academic dishonesty. Model 1.1 – Full Sample. ^∗∗∗^*p* < 0.001.

Figures 2, 3 offer schematic representations of the two models (perceptions and behaviors, respectively) along with standardized estimates using the full sample (i.e., 2012 and 2017 combined; *N* = 1388). As depicted, all items loaded significantly onto their respective factors for both models (λ ’s = 0.51 to 1.07 for Perceptions and 0.72 to 0.86 for Behaviors). As hypothesized, the intercorrelations among the three factors in both models were statistically significant (all *p*’s < 0.001).

### Multi-Group Confirmatory Factor Analysis

Having established that the models for both cohorts had equivalent path configurations with respect to the two three-factor models, MGCFA was conducted to progressively test both models of metric, scalar, and then residual invariance. As detailed in Table 3, based on change (Δ) in CFI, RMSEA, and SRMR values, the three-factor model for perceptions achieved the level of residual invariance – also known as “strict” invariance – and the three-factor model achieved the level of a scalar (or “strong”) invariance. In the latter case, the decision to “reject” invariance of residuals was based on the high change in CFI (Δ = 0.016). Importantly, however, only measurement invariance at the configural, metric, and scalar levels (not the residual level) is necessary to infer the model equivalence and proceed with between-cohort comparisons on the latent factors.

**TABLE 3:** Results from tests of measurement invariance based on cohort.

Model	CFI	RMSEA	SRMR	Δ CFI	Δ RMSEA	Δ SRMR	Decision
**3-Factor Model of Perceptions**							
Model 1 – Configural Invariance	0.975	0.041	0.051	–	–	–	–
Model 2 – Metric Invariance	0.973	0.041	0.050	0.002	0.000	0.001	Accept
Model 3 – Scalar Invariance	0.973	0.038	0.052	0.000	0.003	−0.002	Accept
Model 4 – Residual Invariance	0.971	0.037	0.053	0.002	0.001	−0.001	Accept
**3-Factor Model of Behaviors**							
Model 1 – Configural Invariance	0.979	0.050	0.026	–	–	–	–
Model 2 – Metric Invariance	0.976	0.050	0.025	0.003	0.000	0.001	Accept
Model 3 – Scalar Invariance	0.971	0.051	0.038	0.005	−0.001	−0.013	Accept
Model 4 – Residual Invariance	0.955	0.059	0.030	**0.016**	−0.008	0.008	Reject

*N = 1388. Bold-face value exceed recommended limit of0.015.*

### Descriptive Statistics of the Six Latent Factors

Full sample descriptive statistics (i.e., means, standard deviations, Cronbach’s alphas, potential and actual range of responses, and skew) of the six latent factors measured in this study are detailed in Table 4. Two details are most notable. First, although Cronbach’s alphas varied widely among the six factors – from a low of 0.70 for *Assignment Cheating* and a high of 0.90 for *Test or Exam Cheating* – all were either acceptable, good, or very good. Second, the three factors related to personal engagement in academic dishonesty were significantly skewed (where skew = skewness/standard error of skewness, and values >5.0 are considered large). In other words, for these three factors, a significantly greater proportion of participants used the first two points of the five-point scale (i.e., *Never* and *Once or twice this year*) relative to latter end of the scales (i.e., *About weekly* and *Almost daily*). This was not unexpected as significant positive skews are typical on the AMIS (e.g., Stephens, 2018) as well as other measures of academic dishonesty (e.g., Anderman et al., 1998).

**TABLE 4:** Descriptive statistics for the six latent factors.

				Range		
Variable	*M*	SD	α	Potential	Actual	Skew
AI Policy USE	3.55	0.67	0.76	1–5	1.0–5.0	−2.73
Peer Disapproval of AD	3.36	1.00	0.78	1–5	1.0–5.0	−4.85
Peer Engagement in AD	3.29	0.99	0.84	1–5	1.0–5.0	−2.59
Assignment Cheating	1.69	0.75	0.70	1–5	1.0–5.0	21.03
Plagiarism	1.61	0.78	0.85	1–5	1.0–5.0	23.21
Test or Exam Cheating	1.16	0.39	0.90	1–5	1.0–5.0	68.03

*N = 1388; AI, academic integrity; USE, understanding, support, and effectiveness; AD, academic dishonesty.*

### Bivariate Correlations Among Latent Factors by Cohort

As detailed in Table 5, most of the intercorrelations among the six latent factors were statistically significant. The magnitude of the associations, however, were often small (*r* < 0.30). That is, with the exception of the three types of academic dishonesty – assignment cheating, plagiarism, and test or exam cheating (*r*’s = 0.36 to 0.48, *p* < 0.001). Importantly, the pattern of results (i.e., the direction and strength of the correlation coefficients) were very similar for the two cohorts – all differences were < 0.10 with the exception of the association between assignment and test cheating (*r* = 47 in 2012 and 0.36 in 2017). Finally, as evidenced in the last column and row, the Cronbach’s alphas for all six latent factors were also very similar across the two cohorts.

**TABLE 5:** Intercorrelations among the six latent factors and Cronbach’s alphas by cohort.

Factor	1	2	3	4	5	6	α
1. AI Policy USE	–	0.24***	−0.20***	−0.12**	−0.13***	−0.14***	0.74
2. Peer Disapproval of AD	0.21***	–	−0.12**	−0.30***	−0.27***	−0.24***	0.78
3. Peer Engagement in AD	−0.26***	−0.18***	–	0.22***	0.07*	0.10**	0.83
4. Assignment Cheating	−0.17***	−0.25***	0.20***	–	0.43***	0.47***	0.70
5. Plagiarism	−0.12**	−0.15***	−0.15***	0.48***	–	0.48***	0.85
6. Test or Exam Cheating	–0.08	−0.12**	0.08*	0.36***	0.47***	–	0.90
α	0.77	0.80	0.82	0.69	0.85	0.89	

Intercorrelations and Cronbach’s alphas (α) for the 2012 Cohort (*n* = 780) are presented above the diagonal, and intercorrelations and α for the 2017 Cohort (*n* = 608) are presented below the diagonal. AI, academic integrity; AD, academic dishonesty; USE, understanding, support, and effectiveness. *p < 0.05, **p < 0.01, ***p < 0.001.

### Hypothesis Testing

A series of ANOVAs were conducted to test the study’s four hypotheses. As detailed in Table 6, results offered mixed support. Contrary to the first hypothesis, the level of perceived understanding, support for, and effectiveness (USE) of university policies related to academic integrity was significantly *lower* (not higher) for the 2017 Cohort (i.e., participants who completed the AIC) compared to the 2012 Cohort (*M*’s = 3.44 and 3.66, respectively). Also contrary to hypotheses, there was no significant between-cohort difference in peer disapproval of academic dishonesty and the level of perceived peer engagement in academic dishonesty was significantly *higher* (not lower) for the 2017 Cohort compared to the 2012 Cohort (*M*’s = 3.55 and 3.09, respectively). As hypothesized, participants in the 2017 Cohort reported significantly lower levels of engagement in both assignment cheating and plagiarism compared to participants in the 2012. Cohort; however, there was no significant difference in test/exam cheating. Finally, as indicated by the partial η^2^ values, all of the observed differences were small in magnitude (η^2^ values < 0.06).

**TABLE 6:** Tests of hypotheses: results from ANOVA comparing cohorts.

	2012 Cohort		2017 Cohort				
Variable	*M*	SD	*M*	SD	Δ Mean	*F*(1,1386)	Partial η^2^
AI Policy USE	3.64	0.66	3.44	0.67	−0.20	31.49***	0.022
Peer Disapproval of AD	3.39	0.99	3.32	1.00	−0.07	1.75	0.000
Peer Engagement in AD	3.09	1.00	3.55	0.92	0.46	77.94***	0.053
Assignment Cheating	1.74	0.78	1.63	0.70	−0.11	6.84**	0.005
Plagiarism	1.64	0.81	1.56	0.75	−0.08	3.94*	0.003
Test or Exam Cheating	1.18	0.42	1.14	0.36	−0.04	2.79	0.002

2012 Cohort *n* = 780; 2017 Cohort *n* = 608; AI, academic integrity; USE, understanding, support, and effectiveness; AD, academic dishonesty. *p < 0.05, **p < 0.01, ***p < 0.001.

In order to examine more closely the between-cohort differences in academic dishonesty, each of the eight behaviors was dichotomized (where 0 = *Never did it* and 1 = *Did it at least once*) and cross-tabulated. As detailed in Table 7, Chi-square analyses indicated significant between-cohort differences on three of the eight behaviors. With respect to assignment cheating, compared to the participants in the 2012 Cohort, significantly fewer participants in the 2017 Cohort reported that they had “copied another student’s work and submitted it as their own” (34 to 26%, respectively; a 23.5% reduction). A similar reduction (from 40.4% in 2012 to 33.6% in 2017; a 16.8% reduction) was observed for conventional plagiarism – “From a book, magazine, or journal (not on the Internet): Paraphrased or copied a few sentences or paragraphs without citing them in a paper you submitted.” There was no corresponding decrease for digital plagiarism, which nearly half of all students from both cohorts reported doing at least once in the past year.

**TABLE 7:** Participants self-reported engagement in academic dishonesty by cohort.

	2012 Cohort		2017 Cohort		χ^2^ (1, *N* = 1388)	
Type of Academic Dishonesty	Con	Digital	Con	Digital	Con	Digital
**Assignment Cheating**						
Copied another’s work	**34.0%**	–	*26.0%*	–	10.29***	–
Unpermitted collaboration	62.8%	–	62.2%	–	0.62	–
**Plagiarism**						
Plagiarized a few sentences	**40.4%**	47.7%	*33.6%*	47.1%	6.94**	0.05
**Test or Exam Cheating**						
Used unpermitted notes	**8.5%**	6.2%	*5.6%*	4.3%	4.17*	2.37
Copied from someone else	12.8%	5.1%	12.0%	4.8%	0.21	0.09
Overall	79.4%		77.5%			

Con = Conventional; Overall = percent of students who reported engagement in at least one of the eight behaviors surveyed. Given marginal frequencies, bold-faced percents are higher than expected compared to *italicized* percents. 8*p* < 0.05, **p < 0.01, ***p < 0.001.

The third (statistically) significant difference also concerned a decrease in a conventional form of dishonesty but not one in its corresponding digital analog. Specifically, compared to the participants in the 2012 Cohort, significantly fewer participants in the 2017 Cohort reported that they had used “unpermitted notes or textbooks during a test or exam” (8.5 to 5.6%, respectively; a 34.1% reduction). However, although not *statistically* significant, compared to 2012 participants, a lower percentage of 2017 participants reported using “unpermitted electronic notes (stored in a PDA, phone or calculator) during a test or exam” (6.2 to 4.3%, respectively; a 30.6% reduction). Finally, it’s worth noting that nearly four out of five participants (regardless of cohort) reported engaging at least once in at least one of eight behaviors described.

Finally, ANOVA used to determine if participants (from the 2017 cohort) who completed the AIC more recently (i.e., relative to the completion date of the survey) reported significantly different perceptions or behaviors compared to other participants. As detailed in Table 8, there was no significant differences on any of the six factors based on time elapsed between participants’ completion of the survey and the AIC. In short, contrary to the fifth hypothesis, participants who had completed the AIC within the past 3 months did not report greater understanding or peer disapproval nor lower levels of perceived peer or personal engagement in academic dishonesty, compared to participants who completed the AIC at any time further in the past (i.e., from four to 6 months or two or more years ago).

**TABLE 8:** Results from ANOVA based on time elapsed since participants’ completion of the AI course.

		<3 months	4 to 6 months	7 to 12 months	1 to 2 years	2 or more years	
Variable		(*n* = 38)	(*n* = 78)	(*n* = 70)	(*n* = 222)	(*n* = 197)	*F* (4,604)
AI Policy USE	*M*	3.55	3.41	3.46	3.46	3.39	0.57
	*SD*	0.64	0.73	0.66	0.68	0.64	
Peer Disapproval of AD	*M*	3.46	3.39	3.16	3.29	3.34	0.78
	*SD*	0.92	0.98	1.13	0.99	0.98	
Peer Engagement in AD	*M*	3.53	3.47	3.53	3.49	3.65	1.05
	*SD*	0.86	0.90	0.97	0.94	0.89	
Assignment Cheating	*M*	1.49	1.52	1.68	1.70	1.61	1.54
	*SD*	0.61	0.77	0.64	0.74	0.66	
Plagiarism	*M*	1.49	1.44	1.54	1.62	1.56	0.34
	*SD*	0.66	0.68	0.70	0.80	0.74	
Test or Exam Cheating	*M*	1.09	1.15	1.17	1.14	1.15	1.04
	*SD*	0.17	0.27	0.48	0.35	0.38	

*N = 605. AI, academic integrity; USE, understanding, support, and effectiveness; AD, academic dishonesty.*

## Discussion

The present study sought to extend the existing research on the effects of online academic integrity instruction on university students’ perceptions of and engagement in academic dishonesty. Previous research had shown such instruction to be effective in increasing knowledge and decreasing cheating, but results from the present investigation offered mixed evidence. Contrary to hypotheses, participants who completed the AIC reported: (1) significantly *lower* (not higher) levels of understanding, support, and effectiveness with respect to the University’s academic integrity policies; (2) statistically *equivalent* (not higher) levels of peer disapproval of academic dishonesty, and; (3) significantly *higher* (not lower) levels of peer engagement in academic dishonesty. However, results related to participants’ personal engagement in academic dishonesty offered partial support for hypotheses – those who completed the AIC reported significantly lower rates of engagement on three of the eight behaviors included in the study (copying another student’s work, conventional plagiarism, and use of unpermitted notes during a test or exam). The effect sizes associated with all differences were small. Finally, contrary to hypotheses, there was no evidence of a recency effect on any of the six latent factors; participant responses did not varyas a function of the time elapsed between completion of the AIC and completion of the survey. In short, to the extent that AIC had any effects on the perceptions and behaviors of those that completed it, they were not always as predicted and always modest.

### Significance of Findings

The present investigation offers some potentially important insights concerning the implementation or delivery of online instruction related academic integrity. While previous research had shown such courses or tutorials to be effective in increasing knowledge (e.g., Curtis et al., 2013; Cronan et al., 2017) and decreasing dishonesty (e.g., Belter and du Pré, 2009; Dee and Jacob, 2010; Owens and White, 2013; Zivcakova et al., 2014), their implementation and assessment was confined to a single course and over a short period of time. The findings of this study suggest that online courses or tutorials may not be effective when delivered outside of or abstracted from a specific course. In other words, when delivered as a stand-alone registration requirement, online academic integrity instruction appears limited (if not ineffective) in changing students’ perceptions and behaviors related to academic integrity.

From a learning transfer perspective (e.g., Perkins and Salomon, 1992), the difference in results between previous studies and the present investigation is not unexpected. Specifically, while the former required only *near transfer* (a demonstration of learning in the same or similar context), the AIC (and others like it) demand *far transfer* (an impact in a context outside of or remote from the context of learning). The idea of far transfer – and disagreement about its possibilities – date back to the early 1900s (cf. Woodworth and Thorndike, 1901; Judd, 1908; for a brief review, see Barnett and Ceci, 2002). This long-running disagreement over far transfer has been sustained for over a century because the empirical evidence for it has also been divided, particularly for knowledge and skills associated with critical thinking and problem-solving (cf. Brown, 1989; Sala and Gobet, 2017). Importantly, research has shown support for far transfer is more likely to occur when participants are provided support such as hints or other cues that prompt recall of the previous learning and its potential of generalization or applicability in the new situation (e.g., Gick and Holyoak, 1980; Halpern, 1998).

Transfer of learning, of course, presupposes that learning occurred in the first instance. Given that none of the hypotheses of the present study were supported (i.e., there were no meaningful difference between participants who did and did not complete the AIC, combined with the absence of any recency effects), it’s not clear how much was actually learned (i.e., processed and stored into memory) from the completing the AIC. That said, much like the research on far transfer, recency effects (themselves a type of near “temporal” transfer, see Barnett and Ceci, 2002) are also aided and amplified by prompts and reinforcement (e.g., Lambert and Yanson, 2017). Jones et al. (2006), for example, provided evidence of a “decisional recency effect,” whereby individuals weigh “recent information more heavily, which produces a tendency to choose responses or actions that have recently been reinforced” (p. 316). In retrospect, given the context in which the AIC completed (as a registration requirement and not integrated into any academic course or program of study), the failure to find support for this studies hypotheses is not so surprising (even if somewhat disappointing).

With this in mind, the null findings of this study do not mean that the AIC is useless and unnecessary, but rather that is insufficient when delivered independent of any other efforts to strengthen and support the use of the principles and skills it hopes to teach. Such efforts would include requiring (or at least) encouraging university instructors to reference the AIC in their course, not only to remind students of the principles and practices associated with academic integrity but to offer specific guidance on how those principles and practices are relevant to their course. More generally, as argued elsewhere (e.g., Bertram Gallant, 2011; Wangaard and Stephens, 2011; Seider, 2012; Alzona, 2014; Stephens, 2015, 2019), more holistic approaches are needed to create a culture of integrity and academic dishonesty. One-off interventions, however, well-designed, are unlikely to ameliorate the long-standing epidemic of academic dishonesty.

### Limitations and Future Directions

The present study used an observational research design, a “natural experiment” without true random assignment or control over the administration of the intervention. While convenient and cost-effective, this research design presents limits with respect to making firm causal claims (Campbell and Fiske, 1959). Accordingly, where possible, future studies should use longitudinal or (true) experimental designs to assess with more certainty the effects of interventions such as the AIC. Secondly, these future studies should assess for more and/or different potential outcomes. The present study measured only a handful of *perceptions* and *self-reported* behaviors related to academic integrity. Future research should include assessment of other factors such as future behavioral intentions as well as *actual* knowledge gains associated with completing courses like the AIC and the effects of those gains on *demonstrable* outcomes (including but not limited to academic dishonesty).

## Conclusion

The results of the present study suggest that short, stand-alone courses on academic integrity have only modest, if any, effects on students’ perceptions and behavior. While previous research has shown such courses capable of producing substantial reductions in academic dishonesty (plagiarism, in particular), these courses were required in the context of a specific course. The AIC, in contrast, was not linked to any specific course or program, and its underwhelming effects likely caused by a failure to transfer. The findings suggest that requiring students to complete a course like the AIC may be useful, but only as part of a more comprehensive approach to promoting academic integrity.

## Data Availability Statement

The raw data supporting the conclusions of this article will be made available by the authors, without undue reservation.

## Ethics Statement

The studies involving human participants were reviewed and approved by the University of Auckland Human Participants Ethics Committee in 2012 (Reference Number 2009/C/026) and again in 2017 (Reference Number 019881). The patients/participants provided their written informed consent to participate in this study.

## Author Contributions

JS: conceptualization, formal analysis, writing – original draft of methods, results, discussion, writing. review, and editing of complete manuscript. PW: project administration, writing – original draft of literature review, writing, review, and editing of complete manuscript. MA: project administration, data entry, and formal analysis. GL: data entry, research for literature review, and writing – original draft of literature review. ST: data entry and research for literature. All authors contributed to the article and approved the submitted version.

## Conflict of Interest

The authors declare that the research was conducted in the absence of any commercial or financial relationships that could be construed as a potential conflict of interest.

## Footnotes

1 https://www.wm.edu/offices/deanofstudents/services/communityvalues/honorcodeandcouncils/honorcode/index.php

2 https://spark.library.yorku.ca/academic-integrity-module-objectives/

3 https://www.auckland.ac.nz/en/students/forms-policies-and-guidelines/student-policies-and-guidelines/academic-integrity-copyright/academic-integrity-course.html

## Appendix A

Topical Outline of The University of Auckland’s Academic Integrity Course

Module 1: Academic integrity at the University

1.1 What is academic integrity?
1.2 Understanding the academic environment
1.3 The University of Auckland Graduate Profiles

Module 2: Avoiding academic dishonesty

2.1 Examples of academic dishonesty
2.2 When working in groups…
2.3 When getting and giving help…

Module 3: Using and acknowledging the work of others

3.1 Quoting, paraphrasing and summarizing
3.2 Citing and referencing
3.3 Avoid plagiarism

Module 4: Using copyrighted material correctly

4.1 What is copyright?
4.2 How to use copyrighted material?
4.3 What is Creative Commons? Intellectual property (IP)

Module 5: Consequences of academic dishonesty at university

5.1 What happens if someone is academically dishonest?
5.2 What happens if someone cheats during an exam?
5.3 Equip yourself

## Appendix B

Assignment Instructions Provided to Students (abbreviated)

The purpose of this assignment is to provide you with an opportunity to learn about research methods and reporting practices as well as to deepen your understanding related to some of theories we have been (or will be) reading about and discussing in this course. To do so, we have decided to structure this assignment around the principles of project-based learning and social constructivism more broadly (e.g., active participation, self-regulation, social interaction and construction of knowledge). The project explored in this assignment concerns academic dishonesty – a serious problem in schools and universities around the world (e.g., Murdock et al., 2016). We have created a shortened form of a questionnaire (the AMIS-SF) comprising “measures” related to academic motivation and moral judgment that have been in the research literature to investigate the problem of academic dishonesty (Anderman et al., 1998; Murdock et al., 2004; Stephens and Gehlbach, 2007; Stephens and Nicholson, 2008; Stephens et al., 2010). As indicated above, this Assignment has two parts.

You are asked to recruit a total of [8 in 2012, 15 in 2017] UOA students from at least two different faculties) to complete the AMIS-SF. This questionnaire comprises approximately 30 items that measure various perceptions and behaviors related to academic integrity. These topics are covered in your textbook and will be discussed in lectures 3 and 7 during the semester. In addition, you will also receive readings and in-course training related to the research procedures (e.g., when, where, and how to approach potential participants) and ethical principles (e.g., autonomy, confidentiality, and non-maleficence) inherent in conducting survey research. These procedures and principles are of the utmost importance in conducting “good” research; research that not only produces valid and reliable data but also demonstrates respect for study participants throughout the research process.

Below is a summary of the procedures and principles you must follow when carrying-out this part of the assignment:

• All potential participants must be approached in public spaces on or around the UOA city campus.
• As you approach potential participants, please introduce yourself and state your purpose clearly:
  ∘ *Hello, my name is ______ and I’m conducting some research for an assignment in my educational psychology course. If you’re student at the UOA, like me, I’m hoping you might have a few minutes to complete an anonymous survey. Here* [handover PIS] *is an information sheet detailing the study and your rights as a participant*. [Allow time to read]. *Are you willing to participant?*
  ∘ [If no] *No worries, thanks for considering it. Have a great day* [move on].
  ∘ [If yes]. *Thanks for your willingness to participant. Here’s the Questionnaire* [hand it over, along with the sealable envelope and a pen, if needed]. *It should take approximately 5 min to complete, please remember that the survey is anonymous, so please do not write your name or any identification numbers on it or the envelope. Please also recall that your participation is voluntary, and you may stop at any time without given reason. After you’ve completed the survey, please place it in the envelope and seal it before returning to me. Please let me know if you have any questions.*
  ∘ NOTE: Please give the participant time and space to enter responses privately – do not attempt to look at their response, and only receive questionnaires once they have been sealed in the envelope.
• After you have collected the sealed envelope, it should be mixed amongst the other envelopes you’ve collected and remained sealed until all Questionnaires are collected.
